# Supplementary material for: Rapid turnover of life-cycle-related genes in the brown algae
Source: Genome Biol. 2019 Feb 14;20:35. doi: 10.1186/s13059-019-1630-6 (PMC6374913; doi:10.1186/s13059-019-1630-6)
Supplement: Supplementary file 3 — Protein alignments, corresponding Gblocks html file and CDS sequences for the lineage conserved gametophyte-biased genes. (ZIP 2109 kb) [file 13059_2019_1630_MOESM3_ESM.zip › Con_GA_alignments/SJ21517.aa_ali.fasta-gb.htm]

SJ21517.aa.fasta.trans.aa\_ali.fasta


## Gblocks 0.91b Results

Processed file: **SJ21517.aa.fasta.trans.aa\_ali.fasta**  
Number of sequences: **2**  
Alignment assumed to be: **Protein**  
New number of positions: **222** (selected positions are underlined in blue)

```
                         10        20        30        40        50        60
                 =========+=========+=========+=========+=========+=========+
SJ21517          MGFGHLENAVPLGEELSLEVFDANVPEVLENLRTTQQPGTSQEQCVKNLMSKFLLEELPD
Mac.63601.1      ------------------------------------------------------------
                                                                             


                         70        80        90       100       110       120
                 =========+=========+=========+=========+=========+=========+
SJ21517          LQQGGRHEDVPNATIDAGFIPAAPAATSPTPTPPEMASDGKESDTAVPGERTSSNPSQPR
Mac.63601.1      ------------------------------------------------------------
                                                                             


                        130       140       150       160       170       180
                 =========+=========+=========+=========+=========+=========+
SJ21517          RLNAGKAPAAAGGGSEGAGGGGSSTSSSASNASSGRAGCPSGSGGSSSGSGSGGGGGGGG
Mac.63601.1      ------------------------------------------------------------
                                                                             


                        190       200       210       220       230       240
                 =========+=========+=========+=========+=========+=========+
SJ21517          GGGGGGSVFCDVASAGSAGLVMSSPVDLEPILLPASSSSLLSAASPTFDAAAAAPPPEKG
Mac.63601.1      ------------------------------------------------------------
                                                                             


                        250       260       270       280       290       300
                 =========+=========+=========+=========+=========+=========+
SJ21517          AAGSSAYTAMTAANGSGGAAPVPYPPIGQGALPSRYPSGNGDDRLWLVYDGVSAAGKGVI
Mac.63601.1      ------------------------------------------------------------
                                                                             


                        310       320       330       340       350       360
                 =========+=========+=========+=========+=========+=========+
SJ21517          SKVNAPDPYWLGRASPGVVCPHAHSMEFHSSFESANLLRAVQRGPAEYDIFLRPDLHTHG
Mac.63601.1      ------------------------------------------------------------
                                                                             


                        370       380       390       400       410       420
                 =========+=========+=========+=========+=========+=========+
SJ21517          HTQWFYFAVRDTHPPGMLGESVVKFNVINLTKPDSLFAMGMRPVLYSHQDAAEKGLGWRR
Mac.63601.1      ------------------------------------------------------------
                                                                             


                        430       440       450       460       470       480
                 =========+=========+=========+=========+=========+=========+
SJ21517          CGHGIDYRSNGYSWYKPDGSQASFYTLSLSVTFSNPGDTYRLAHAHPYSYSDHKRHLAKL
Mac.63601.1      ------------------------------------------------------------
                                                                             


                        490       500       510       520       530       540
                 =========+=========+=========+=========+=========+=========+
SJ21517          LADERTAKFLHHTVLCTTVGGNECDLLTITDSKEAPITATGNGKSDTGGPSHPSTPDLGQ
Mac.63601.1      ------------------------------------------------------------
                                                                             


                        550       560       570       580       590       600
                 =========+=========+=========+=========+=========+=========+
SJ21517          QQQQQQQHEGYLLGTNGVRYTNRERPEPASVGEGVAAVVAAAAAAAVADGGDIARGGGEG
Mac.63601.1      ------------------------------------------------------------
                                                                             


                        610       620       630       640       650       660
                 =========+=========+=========+=========+=========+=========+
SJ21517          TVNGAGDLLGIGGASSGFGGAVGGGAGWDGDAGAGAGASSRRGSHKRCVVISARVHPGET
Mac.63601.1      ------------------------------------------------------------
                                                                             


                        670       680       690       700       710       720
                 =========+=========+=========+=========+=========+=========+
SJ21517          PASWMMRGMLDFITGESAEARLLRSLFVFKIVPMLNPDGVAYGNNRCSLAGVDLNRQWKR
Mac.63601.1      ------------------------------------------------------------
                                                                             


                        730       740       750       760       770       780
                 =========+=========+=========+=========+=========+=========+
SJ21517          PSRALHPTVFWLKQHIRQEQAKRGVAMYVDLHGHSRKMNIFMYGADEKRRGLSCPSARVF
Mac.63601.1      ------------------------------------------------------------
                                                                             


                        790       800       810       820       830       840
                 =========+=========+=========+=========+=========+=========+
SJ21517          PKLLSWNRLGRKYVSFKDCSFAVKKGRESTARVVVARDLGIGNSYTVESTFCGVDFGPLK
Mac.63601.1      ------------------------------------------------------------
                                                                             


                        850       860       870       880       890       900
                 =========+=========+=========+=========+=========+=========+
SJ21517          DHHLNTDHLQEAGVAICDALLDYYLPNQAQRERAHRDLMARDGVAEERGGGLTVARPTII
Mac.63601.1      ------------------------------------------------------------
                                                                             


                        910       920       930       940       950       960
                 =========+=========+=========+=========+=========+=========+
SJ21517          APAVGRISSEGGRDGGSGGSDSCCARGDENERRQDGEARDVDDDVVDNNDDIASEDDNDS
Mac.63601.1      ------------------------------------------------------------
                                                                             


                        970       980       990      1000      1010      1020
                 =========+=========+=========+=========+=========+=========+
SJ21517          EPPEISPGNADADPDTDLATLSPRHTPPDVGKGPNTRPSIESGITGQPFDVGTRLVLSGR
Mac.63601.1      ------------------------------------------------------------
                                                                             


                       1030      1040      1050      1060      1070      1080
                 =========+=========+=========+=========+=========+=========+
SJ21517          VSGSGGNRGGSDRAAENTAGVGGRAREECDESHGDRMGNERGRGWGLQTVGGHVGDGGGG
Mac.63601.1      ------------------------------------------------------------
                                                                             


                       1090      1100      1110      1120      1130      1140
                 =========+=========+=========+=========+=========+=========+
SJ21517          DKVGGCEGEGHPWLGVGWEQGEVGGSGEAVRRWGRAVKEVDDGDDESAIESAELEGLAIE
Mac.63601.1      ------------------------------------------------------------
                                                                             


                       1150      1160      1170      1180      1190      1200
                 =========+=========+=========+=========+=========+=========+
SJ21517          QQPPAGGQPAGKRVELRRGDSVKYAGGAAIGADGNRWTGRGKGCRRSSTRERGSGGASGA
Mac.63601.1      ------------------------------------------------------------
                                                                             


                       1210      1220      1230      1240      1250      1260
                 =========+=========+=========+=========+=========+=========+
SJ21517          EAIAPVAVSTSCTAAAAGGNPTGGEPPPSGRRKEKRRGSGSRNAKHLALTNGNRGKGSDR
Mac.63601.1      ------------------------------------------------------------
                                                                             


                       1270      1280      1290      1300      1310      1320
                 =========+=========+=========+=========+=========+=========+
SJ21517          TTAIRQHRPRTHGNGGDGAARRASDGAIQAASSVLAACSSSSTPTSHPRRRIRSLAGGGA
Mac.63601.1      ------------------------------------------------------------
                                                                             


                       1330      1340      1350      1360      1370      1380
                 =========+=========+=========+=========+=========+=========+
SJ21517          SGGGGRFTPDNGNNGGSSSRGGSGSSHSSKGAGGGGGGGRSRGRNGG-GSGSSTHGIVSS
Mac.63601.1      ----------NGNNGGSSSRGGSGSSHSSKGAGGGGGGGRGRGRNGSSGNGSNIHGIVSS
                           ####################################  ############


                       1390      1400      1410      1420      1430      1440
                 =========+=========+=========+=========+=========+=========+
SJ21517          SAAGDLEPAPRSGSSLDARDRGAEEAAAATGVVKRGGSLRWLSPTDPAAVGGAEANWGGM
Mac.63601.1      SAAGDLESAPRSGSSLDARGRGTEEAATATGVVKRGGSLRWLSPTDPTSAGGTEVNWGGM
                 ############################################################


                       1450      1460      1470      1480      1490      1500
                 =========+=========+=========+=========+=========+=========+
SJ21517          GSLPPNFVRRLVPNQSSSTERRGNEARTARQADLGVPEIGNDVARGGGGG-ATGGKLKAM
Mac.63601.1      GSLPPNFVRRLIPNQSPSTERRGNDSRT-RQADLGVPEIGNDVARGGGGGGATSGKLKAT
                 ############################ ##################### #########


                       1510      1520      1530      1540      1550      1560
                 =========+=========+=========+=========+=========+=========+
SJ21517          KG---------GRVVKGRSILVSGVGERGDATTSTTPLLALPVLEEAGGGGGGGGGGGGV
Mac.63601.1      KGGALCGGSGRGRVVKGRSILVSGVGDRGDGTNSTTPMLALPVLED--GGGGGGGGGGGV
                 ##         ##################################   ############


                 
                 ========
SJ21517          VVRGLGGR
Mac.63601.1      VARGLSGR
                 ########
```

```
Parameters used
Minimum Number Of Sequences For A Conserved Position: 2
Minimum Number Of Sequences For A Flanking Position: 2
Maximum Number Of Contiguous Nonconserved Positions: 8
Minimum Length Of A Block: 10
Allowed Gap Positions: None
Use Similarity Matrices: Yes
```

```
Flank positions of the 6 selected block(s)
Flanks: [1331  1366]  [1369  1468]  [1470  1490]  [1492  1502]  [1512  1545]  [1549  1568]  

New number of positions in SJ21517.aa.fasta.trans.aa_ali.fasta-gb:  222  (14% of the original 1568 positions)
```
